# Influenza

**Published:** 1891-07-11

**Authors:** 


### INFLUENZA.

The influenza is an acute infective malady which has from time to time for centuries spread over the whole inhabited world. In occurs in epidemics, often pandemically, and has been observed in all parts of the globe, and at all seasons of the year.

Although a good deal of uncertainty exists as to the exact character of certain epidemics recorded in the centuries immediately preceding and following the commencement of our era, the subjoined account may be received as containing all that is known historically of its ravages. The earliest mention is by Thucydides, B.C. 412. In A.D. 590 or 591 it spread from Italy, France, and Spain, over the rest of Europe, attacking not only men, it is reported, but animals as well. At that epogue the disease was so fatal that when Pope Gregory ordered a Holy Procession in Rome, no less than eighty persons fell suddenly dead in the streets with symptoms of protracted sneezing. Hence originated the old popular custom of exclaiming when one sneezed, "God help you!" Other widespread epidemics are recorded in the year 1386, when not one person in ten escaped; in 1580, when influenza broke out in Rome, Tübingen, and Delft; in 1610; in 1647, both in Europe and America; in 1729, when it passed from Russia over all Europe, and as far as Mexico with lightning rapidity; and in 1782, when it travelled from China over Siberia to all Europe, in St. Petersburg alone 40,000 persons falling ill.

Certain epidemics have been limited to particular regions, among which may be mentioned those of A.D. 827; 876 involving only Italy and a part of Germany along the Rhine; 1327 confined to a part of Italy; 1403, an epidemic confined to Paris, but of very grave character; 1411, also confined to Paris, when more than 100,000 persons fell ill within three weeks; 1655; 1742-4; 1799-1800; 1802-3; 1831 and 1833, the last involving Russia and part of North Germany.

Various names have been given to the complaint. In England it has been termed Influenza or Epidemic Catarrh; in France, La Grippe; and in Germany, Influenza, or, as in 1712, the Fashionable Sickness.

The attack usually ends in entire recovery. Sufferers fall suddenly ill, prodromata such as malaise and nasal irritation being unusual. The onset is characterised by more or less shivering without distinct rigour, much feeling of illness, pains in the limbs and back, headache, and fever. Commonly there is catarrhal affection of the mucous membranes, especially those of the respiratory tract. Cough may be very troublesome. Serious complications, such as inflammation of the lungs, digestive disorders, faints, delirium, and convulsions are rare. In from three to four days all the symptoms abate, and in eight days or a fortnight the patient is generally convalescent. In slight cases the whole illness does not last more than from one to three days; but the feeling of illness is out of all proportion to the other symptoms, leaving the patient for a length of time weak and feeble. The few fatal cases occur either among children, aged persons, or those of debilitated constitution. One attack does not protect in the future, second attacks being observed from time to time in the course of the same epidemic.

The causation of the epidemic is not understood. Some have attributed it to the tides, some to an unhealthy poising of the globe, some to volcanic agency; others to abnormal electrical conditions, and yet others have blamed the heavenly bodies.

It is not certainly known whether the disease is catching from man to man. The epidemics which occur in the course of a pandemic speedily reach a maximum, and spread over wide districts with great rapidity without any connection being discoverable with the ordinary routes of human intercourse. The march of the epidemic has often been in a direction from east to west; often from west to east; sometimes from north to south. Mountain and sea do not appear to oppose any obstacles to its progress; at least, outbreaks have been observed on board ships which have for weeks never come near land. It attacked 144 out of 340 souls on board a Dutch frigate lying in the harbour of Macassar. Whole fleets have been compelled to put back to port in consequence of its appearance. It has broken out on many occasions on ships anchoring off coasts where the disease was raging.

The rate at which it travels from place to place is liable to wide variations, but is mostly astonishing. In the pandemics of 1833 and 1837 it attacked almost simultaneously regions widely separated; at other times it has advanced very tardily. Progress by leaps and bounds is observed now and then, and sometimes changes from the initial direction take place in the course of its extension.

Particular localities or particular classes of the population are sometimes spared in a way which has not been explained. It affects all races indifferently, appears in every climate and at every season; without, however, atiological connection between season and weather and the outbreaks being recognisable.

The term influenza, as is well known, has been applied to certain diseases occurring among the lower animals; but the diseases so-called are no single malady, and although there may be an occasional resemblance to human influenza, any close relationship may be pretty certainly excluded.

This year's epidemic has, so far as the reports at our disposal permit us to judge, advanced with rapid strides, often by leaps and bounds, in the course of a few months, from Russia over the whole continent, breaking out then in England, and last of all in America. Its character has been, on the whole, benign. Suggestions for cure and treatment of the disease are not intended to be offered here. The best course to adopt is to call in seasonable medical advice; but taking into consideration the extension of the epidemic and the injury done thereby to trade and commerce, it may be pointed out what steps are to be taken for hindering the yet wider development of the malady.

Large assemblies of human beings are, where possible, to be avoided. Where unavoidable, as in places of public worship, care must be taken not to catch colds, which appear to predispose to influenza. There should be as little as possible variation in the temperature of rooms and dwellings while the epidemic lasts. Intercourse with the sick must be limited, since it is not certain that contagion does not ocour. Overheating as well as underheating is harmful, the best temperature being 64° Fahr. Let those who have to go into the open air protect themselves as far as possible against the wind. The mouth should be kept closed, and inspiration be effected through the nasal passages.

